# Statistical evaluation of diet-microbe associations

**DOI:** 10.1186/s12866-019-1464-0

**Published:** 2019-05-09

**Authors:** Xiang Zhang, Max Nieuwdorp, Albert K. Groen, Aeiko H. Zwinderman

**Affiliations:** 1 Department of Experimental Vascular Medicine, Amsterdam University Medical Center, Meibergdreef 9, Amsterdam, The Netherlands; 2 Department of Internal and Vascular Medicine, Amsterdam UMC, University of Amsterdam, Meibergdreef 9, Amsterdam, The Netherlands; 3 Department of Clinical Epidemiology, Biostatistics, and Bioinformatics, Amsterdam UMC, University of Amsterdam, Meibergdreef 9, Amsterdam, The Netherlands

**Keywords:** Microbiome, Diet, Association, Simulation, Sequencing

## Abstract

**Background:**

Statistical evaluation of the association between microbial abundance and dietary variables can be done in various ways. Currently, there is no consensus on which methods are to be preferred in which circumstances. Application of particular methods seems to be based on the tradition of a particular research group, availability of experience with particular software, or depending on the outcomes of the analysis.

**Results:**

We applied four popular methods including edgeR, limma, metagenomeSeq and shotgunFunctionalizeR, to evaluate the association between dietary variables and abundance of microbes. We found large difference in results between the methods. Our simulation studies revealed that no single method was optimal.

**Conclusions:**

We advise researchers to run multiple analyses and focus on the significant findings identified by multiple methods in order to achieve a better control of false discovery rate, although the false discovery rate can still be substantial.

## Background

With the help of high-throughput sequencing technologies, human microbiota have been profiled and studied extensively [1]. Since diet shapes the composition of human microbiota and influences human health, linking abundance of microbes to dietary variables is a common practice in human microbiome studies [2, 3]. These association studies not only can improve our understanding of the relationships between the human microbiome and nutrient intake, but also may help development of new therapeutic interventions.

Microbiome data are often generated by targeted sequencing of the 16S ribosomal RNA (rRNA) gene, and represented as a frequency matrix giving the number of times each microbe is observed in each sample. In general microbiome data have following features: 1) library sizes can vary by orders of magnitude across samples. 2) microbiome data often have excess zero counts. These zero counts can be due to either biological absence of a microbe, or insufficient sequencing. 3) microbiome data are compositional data, meaning that the obtained counts do not reflect the absolute number of microbes that are present. 4) microbiome data are over-dispersed, characterized as some taxa (e.g., *Bacteroides* and *Lactobacillus* species) are common among samples, many other taxa are present at much lower abundances.

Various statistical methods have been developed for microbiome data analysis, but there are no standard procedures to perform association analyses [4]. Previous benchmark works [5, 6] focused on case-control studies, and revealed that the choice of statistical methods considerably affected outcomes of differential relative abundance tests. Unlike case-control studies, association studies work also on continuous variables. To our best knowledge, the influence of choosing different methods on outcomes of association studies has not been evaluated. To assess the influence, we analyzed the associations between dietary variables and gut microbiota in 1090 individuals from the HELIUS-cohort study (Amsterdam, the Netherlands) [7, 8]. Since the focus of the current work is on robustness of the statistical results rather than biological or epidemiological associations, biological interpretation of diet-microbe associations is out of the scope of this work. We used four methods including those based on Poisson (shotgunFunctionalizeR), negative binomial (edgeR), zero-inflated Gaussian (metagenomeSeq) distributions, as well as a weighted linear regression model (voom + limma). We compared the results derived from the four methods and observed large differences. To find out which method we should choose in which circumstances, we ran simulation studies and found that no single method was optimal for all microbiome data sets. We advise researchers to run multiple statistical analyses and focus on the significant findings identified by multiple methods in order to achieve better control of false discovery rate.

## Results

### Large difference in results between statistical analyses

**Fig. 1:**
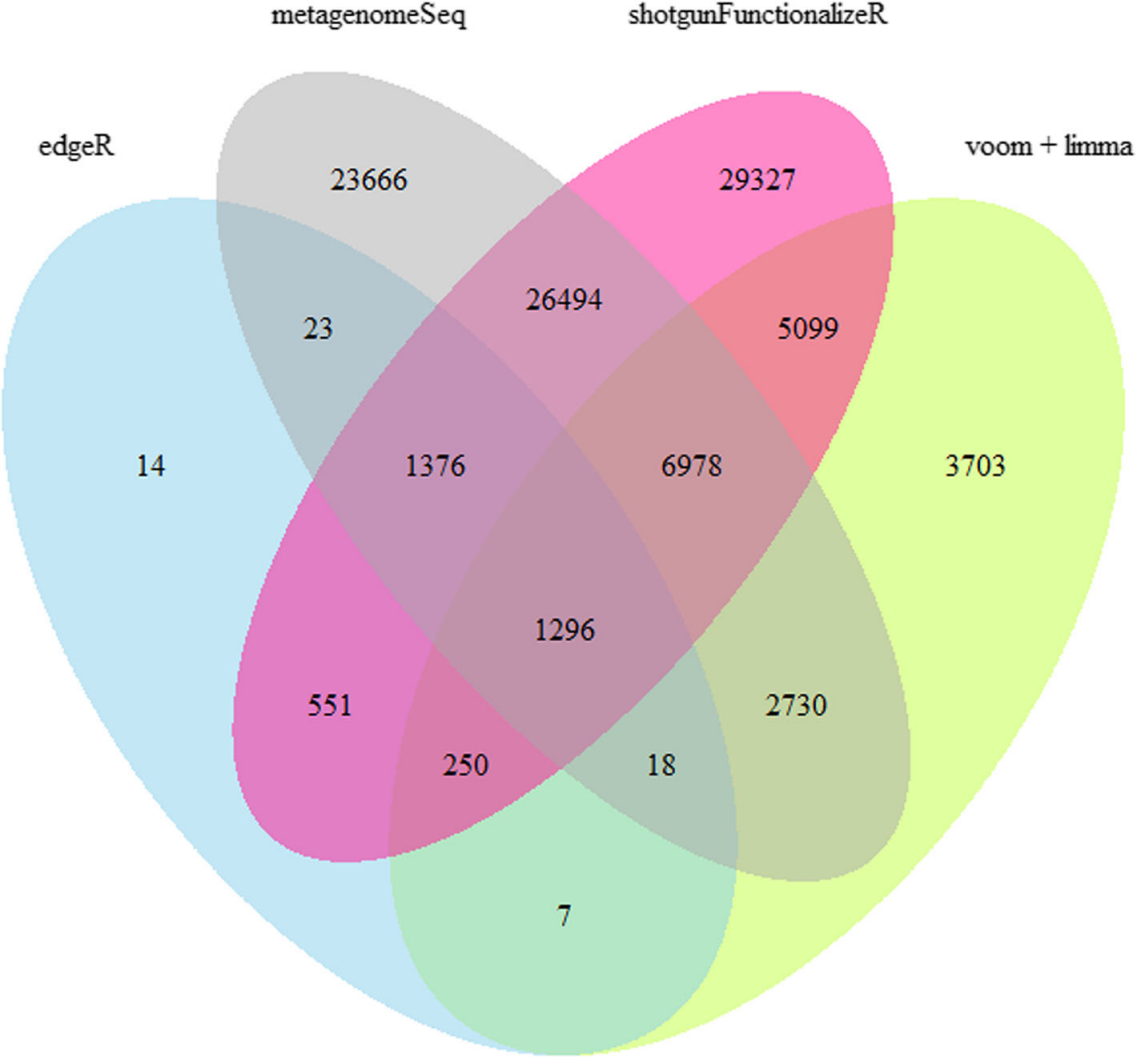
Venn diagram of significant associations identified by edgeR, voom + limma, metagenomeSeq and shotgunFunctionalizeR based on HELIUS 16S rRNA microbiome and FFQ data

To evaluate effect of choosing different methods on outcomes in association studies, we performed association analyses between 67 dietary variables and 2073 OTUs derived from 1090 HELIUS participants with four methods. Out of 138,891 association tests, we identified 3535, 20,081, 62,581 and 71,371 associations with FDR below 0.05 in edgeR, voom + limma, metagenomeSeq and shotgunFunctionalizeR, respectively. There were 1296 associations identified to be significant by all the four methods. In addition, there were 14, 3703, 23,666, and 29,327 associations that were identified as significant only by edgeR, voom + limma, metagenomeSeq or shotgunFunctionalizeR (Fig. 1).

### 16S rRNA microbiome data simulation

**Fig. 2:**
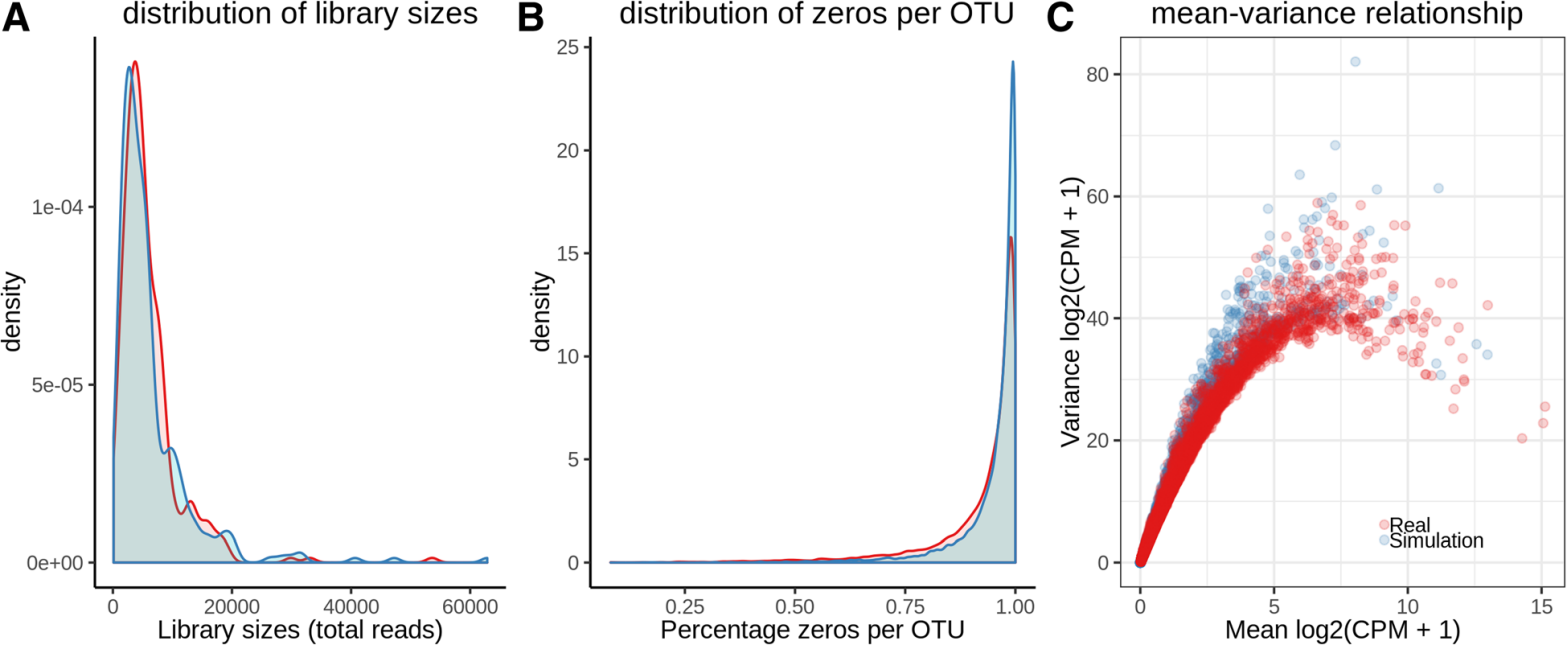
Comparison of simulated and real Human Microbiome Project stool 16S rRNA data. A: library size distributions B. distribution of percentage of zeros per OTU. C. mean-variance relationship. Every dot represents an OTU

**Fig. 3:**
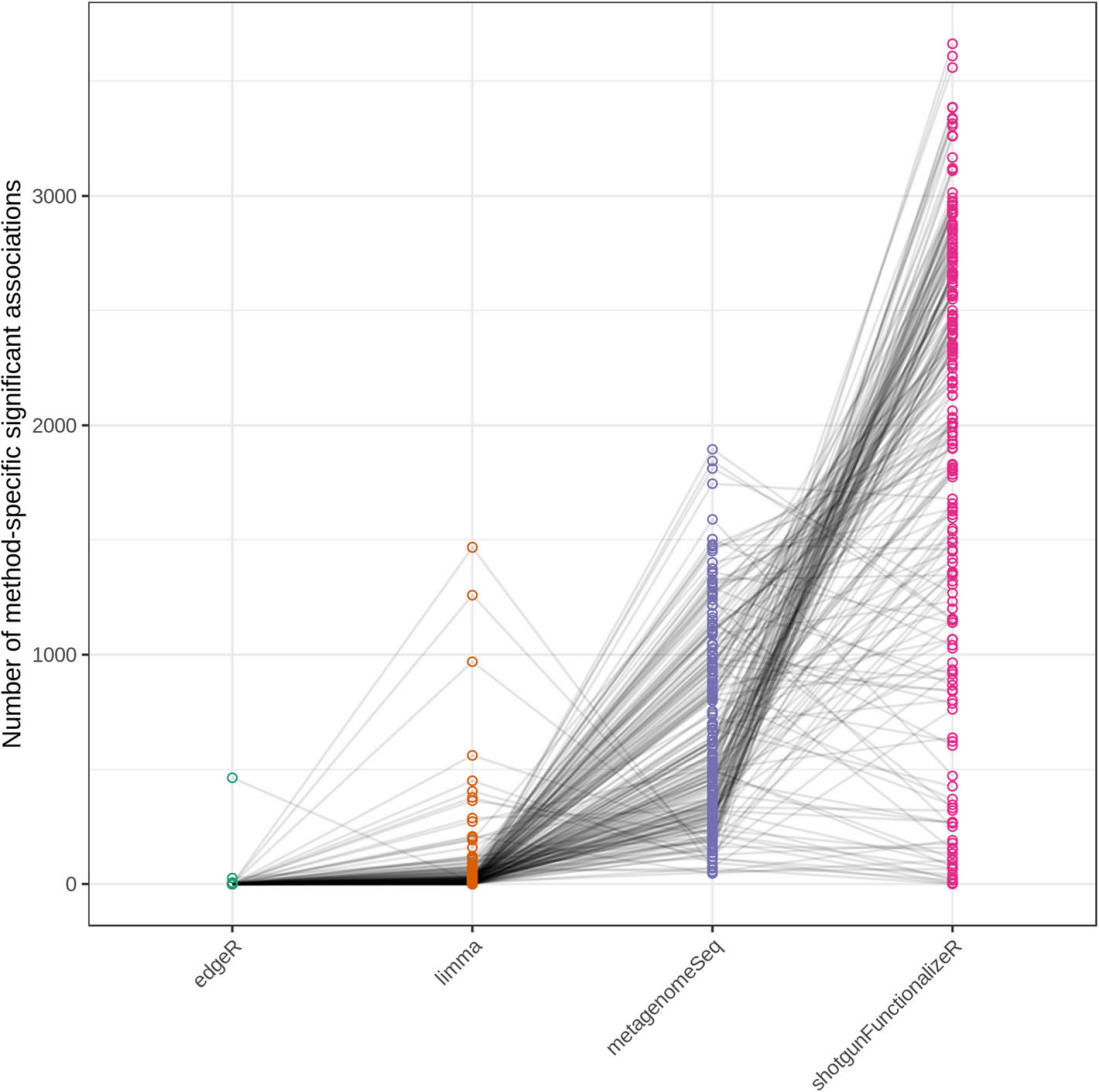
Every dot represents the number of significant associations identified only by the corresponding method. Each line represents a simulation, in which the same simulated data were analyzed by edgeR, voom + limma, metagenomeSeq and shotgunFunctionalizeR

After realizing such considerably different results between the methods, we attempted to find out which method we should choose. To this end, we simulated 16S rRNA microbiome data with spiked-in associations between dietary variables and OTUs. We used a published FFQs (food frequency questionnaires) data as a template. To make sure our simulation framework can generate similar microbiome data as real ones, we compared our simulated data to the real HMP (Human Metabolome Project) stool 16S data. Our simulated microbiome data had similar distribution of library sizes and percentage of zeros per OTU, as well as similar mean-variance relationship (Fig. 2). Our template FFQs data contained 214 dietary variables. In each simulation, we used one dietary variable. Therefore, in total we generated 214 simulated 16S rRNA data sets. Each data set contained 1000 subjects and had mean library size 50,000, and the same simulated data set was analyzed by edgeR, voom + limma, metagenomeSeq and shotgunFunctionalizeR. In our simulations, we observed large difference in results between the methods (Fig. 3).

### Method comparisons based on simulated data

**Fig. 4:**
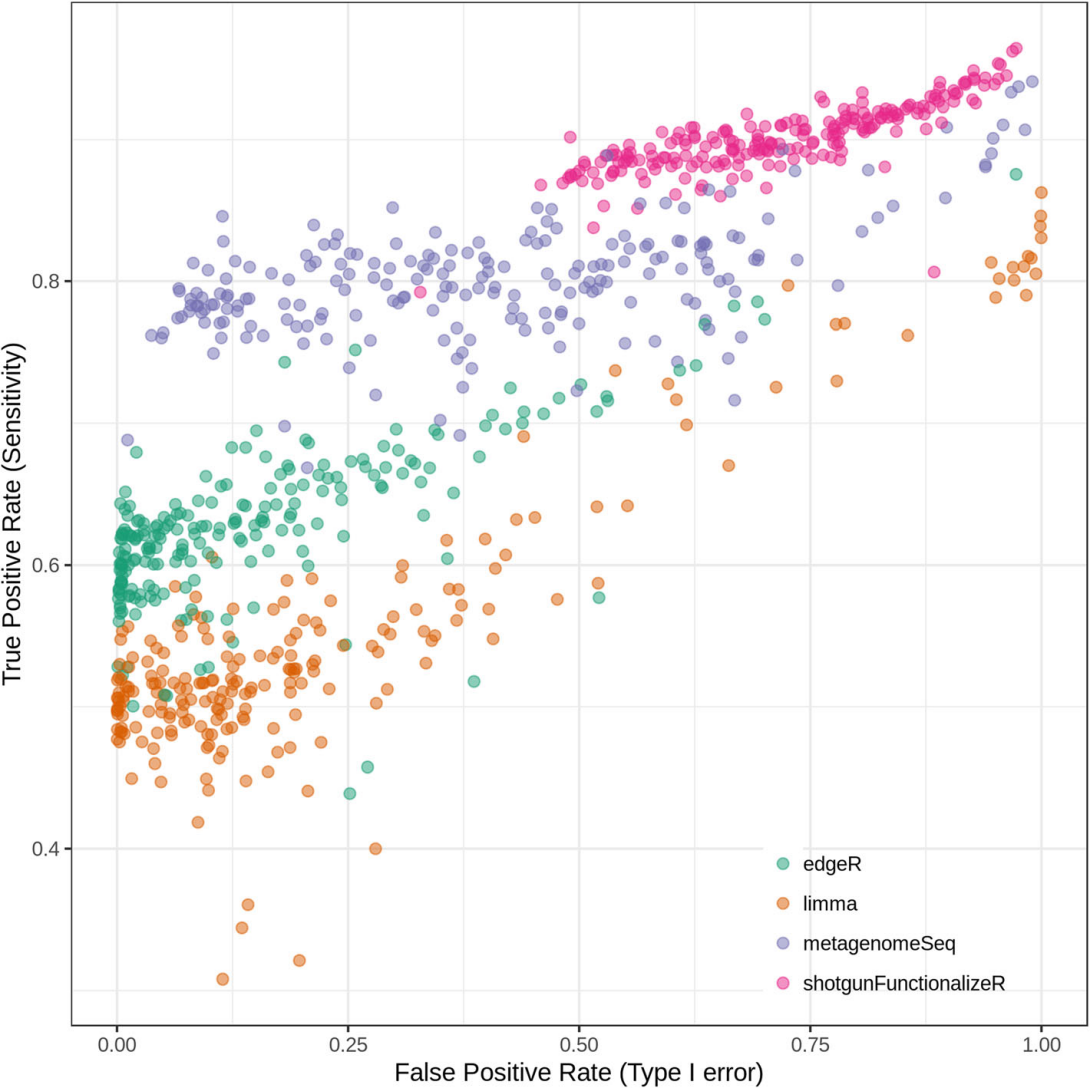
With each simulated data set, we calculated the performance of every method, in terms of true positive rate and false positive rate. Every dot represents a simulation

**Fig. 5:**
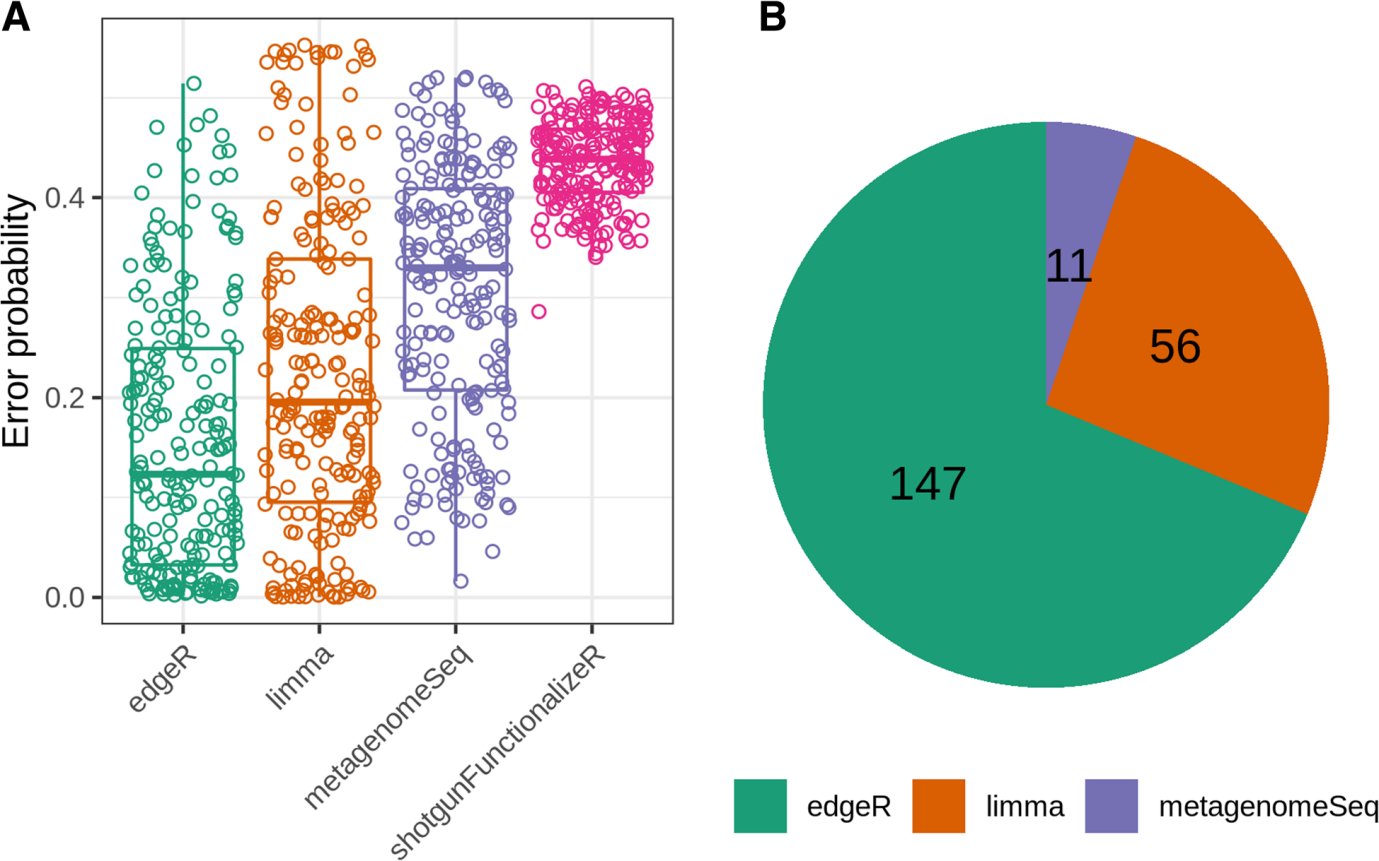
A: Every dot represents the probability that a significant association is false. B: Among 214 simulations, edgeR had the lowest error probability in 147 simulations; limma had the lowest error probability in 56 simulations; metagenomeSeq had the lowest error probability in 11 simulations

**Fig. 6:**
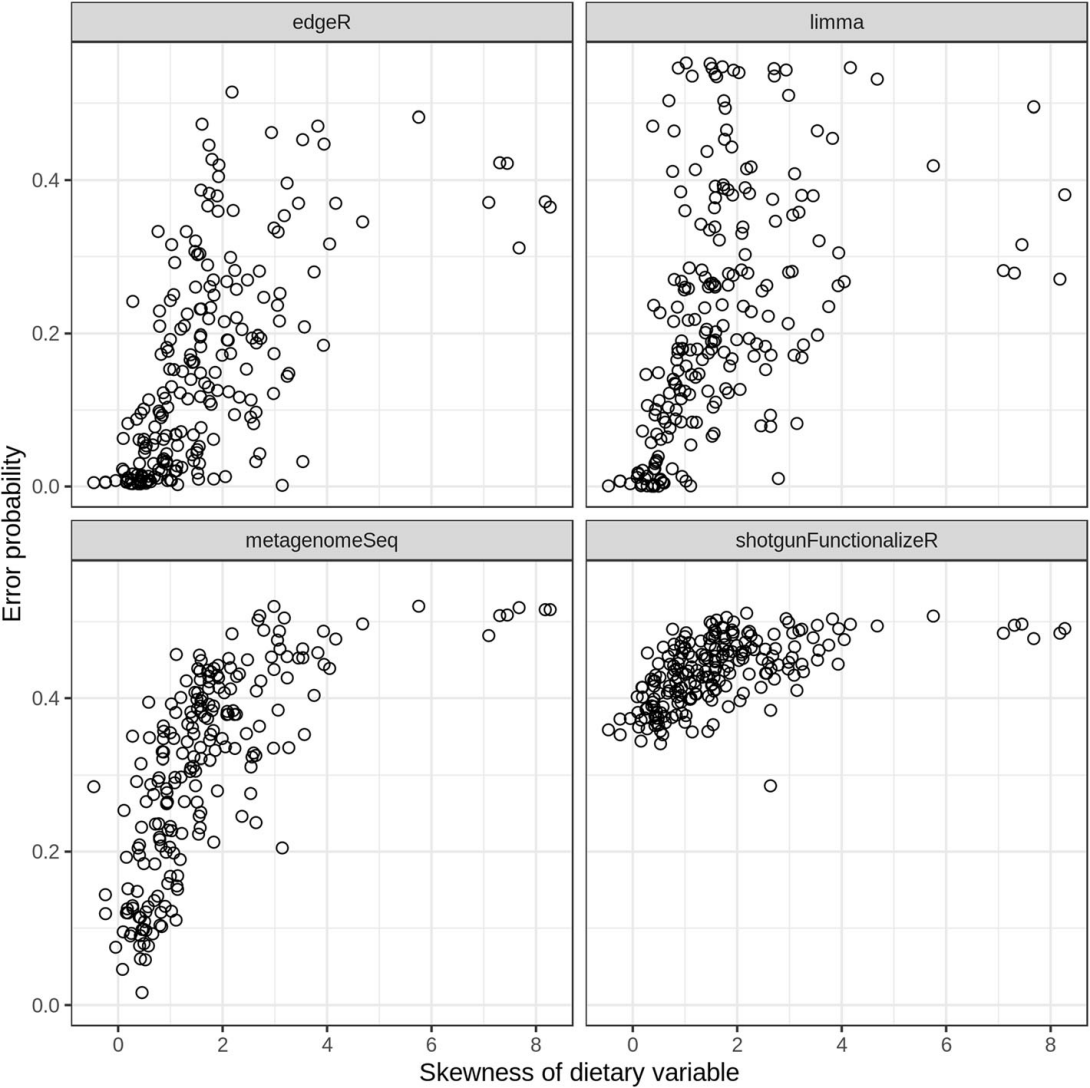
Skewness of predictor variable influences false positive rate. Every circle represents a simulation

**Fig. 7:**
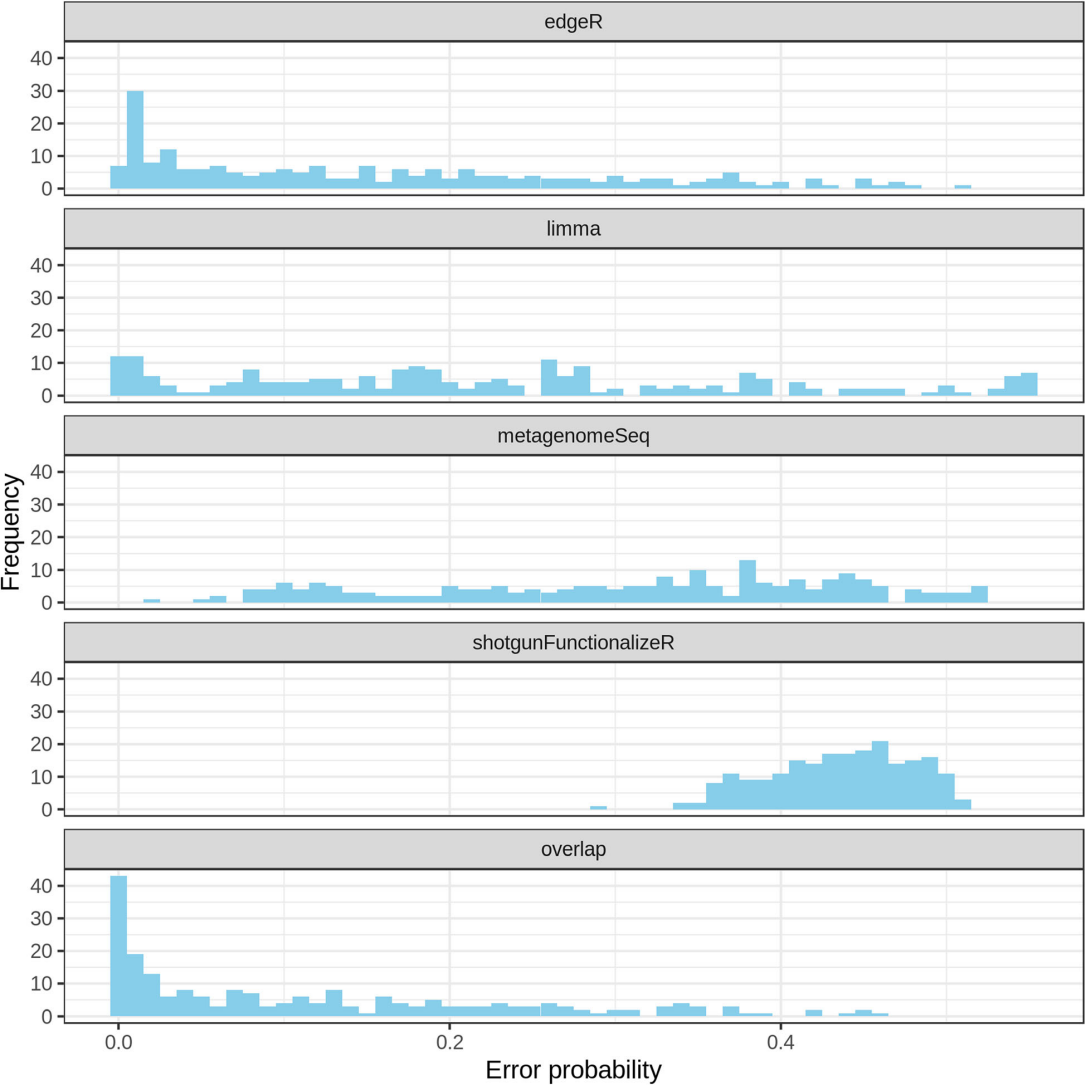
Distribution of probabilities that a significant association is false in edgeR, limma, metagenomeSeq and shotgunFunctionalizeR. The “overlap” refers to the distribution of error probabilities of significant associations identified by all four methods

Overall shotgunFunctionalizeR had both the highest true positive rate and the highest false positive rate (Fig. 4). The median true positive rate of shotgunFunctionalizeR was (0.900), followed by metagenomeSeq (0.800), edgeR (0.624) and limma (0.519). Meanwhile the median false positive rate of shotgunFunctionalizeR, metagenomeSeq, limma, and edgeR were 0.716, 0.388, 0.125 and 0.0898, respectively. Based on the 214 simulations, we identified that the median error probability, defined as the probability that a significant association is false, of shotgunFunctionalizeR, metagenomeSeq, limma and edgeR were 0.439, 0.330, 0.196 and 0.123, respectively (Fig. 5a). Among the 214 simulations, we observed that edgeR had the lowest error probability compared to other methods in 147 simulations, followed by limma (56 simulations) and metagenomeSeq (11 simulations) (Fig. 5b). Furthermore, the error probabilities in different methods were also influenced by the skewness of the distribution of the dietary variables (Fig. 6). In the next step, we identified that 30% simulations in edgeR, 16% simulations in limma, 0.9% simulations in metagenomeSeq and 0% simulation in shotgunFunctionalizeR had error probabilities below 0.05 over the 214 simulations (Fig. 7). However, when we focused on the significant associations that were identified by all four methods (we call them “overlap”) in each simulation, we observed that 44% simulations had error probabilities below 0.05 over the 214 simulations (Fig. 7).

## Discussion

We learned from these relatively simple analyses that a key issue in the analysis of 16S rRNA microbiome data is the choice of the statistical method. Depending on the choice of statistical method, significant associations between dietary variables and microbial abundances varied dramatically. We observed that shotgunFunctionalizeR produced the largest number of unique significant associations, whereas most of the significant associations identified by edgeR were also identified by other methods. What really puzzled us is the relatively small number of significant associations identified by all methods. We think that such dramatic difference is related to the distribution assumptions as well as the normalization processes implemented in the statistical methods [9]. In this study, shotgunFunctionalizeR and edgeR modeled the unnormalized counts with either Poisson or negative binomial distribution, and coped with uneven library size across samples by including the log(total counts) as the offset. In contrast to shotgunFunctionalizeR and edgeR, limma and metagenomeSeq were based on the Gaussian and zero-inflated Gaussian distributions, requiring transforming discrete counts into continuous quantities. To this end, limma transformed the raw counts into log-cpm (log counts per million), in which unequal library sizes were normalized. In metagenomeSeq, uneven library size was normalized by cumulative sum scaling, and the normalized counts were log2 transformed in order to be incorporated into the zero-inflated Gaussian model.

To find out which method we should choose for association studies, we developed a hierarchical model to simulate 16S rRNA data based on dietary variables with spiked-in associations. By comparing to the real HMP 16S microbiome data, we have shown that our simulation model can simulate realistic 16S rRNA microbiome data. Although in this work we focused on diet-microbe association analyses, our simulation framework can easily be adapted to simulate other scenarios.

Based on our simulation model, we generated a large number of 16S microbiome data sets with sample size 1000 subjects and mean of sequencing depth 50,000. These settings were used to mimic the HELIUS data set. When we analyzed the simulated data sets with edgeR, limma, metagenomeSeq and shotgunFunctionalizeR, we observed again large difference in number of significant associations between the methods. In general, we want our statistical method to detect as many as possible true positives, and as few as possible false positives. From our simulation studies, we learned that overall the most sensitive method (shotgunFunctionalizeR in this case) was likely to be the one with the most false positives. This phenomenon was observed in the differential abundance test scenario as well [5]. Even though we set FDR as 0.05 in all our diet-microbe association analyses, our simulation results showed that control of FDR completely failed in shotgunFunctionalizeR, and rarely achieved in metagenomeSeq. On the other hand, edgeR and limma achieved FDR 0.05 in some cases. In the previous case-control simulations [6], metagenomeSeq and shotgunFunctionalizeR were shown to fail controlling false discovery rate at 0.05. However, edgeR was reported to be able to control false discovery rate at 0.05 [6]. We think this is due to the fact that performing association analyses is more challenging than case-control comparisons because we cannot control both dependent and independent variables. Our further analysis showed that the skewness of the independent variable (e.g. dietary variable) influences the error probabilities in all methods. When the skewness of the dietary variable increased, the probability that a significant association is false also increased. When we focused on the significant diet-microbe associations that were identified by all four methods, we observed that more simulations had error probability below 0.05.

## Conclusions

In summary, the choice of the statistical method is a key issue in the analysis of 16S rRNA microbiome data. No single method was optimal for diet-microbe association analyses. We recommend researchers to run multiple statistical models and focus on the significant associations identified by multiple methods. In this way, we can improve the controlling of false discovery rate, although the false discovery rate can still be substantial.

## Methods

### Subjects and HELIUS cohort

Subjects were participants in the HEalthy Life in an Urban Setting (HELIUS) cohort study. This study used a stratified-random sampling approach to include between 2011 and 2015 25,000 inhabitants (18–70 years) from the city of Amsterdam, the Netherlands [7]. Stratification was done on six subgroups with different ethnic origins (African Surinamese, South Asian Surinamese, Ghanaian, Turkish, Moroccan, and Dutch). Subgroups were about equally large.

### Dietary intakes assessment

As described previously [10, 11], a subsample of voluntary participants of Dutch, Moroccan, Turkish, South-Asian Surinamese and African Surinamese origin were asked to participate in the HELIUS-Dietary Patterns study, with objective to collect detailed information on their diet. Usual dietary intakes were assessed through the completion of ethnic-specific semi-quantitative food frequency questionnaires (FFQs) with a reference period of 4 weeks. These FFQs were adapted from an existing Dutch FFQ and comprised about 200 items. Food items were collapsed into 73 food groups based on similarity in nutrient profile and culinary use. In this study ethnic-specific food groups were not included in this analysis and 67 food items were used for the analyses.

### 16S processing

We used the 16S ribosomal RNA (rRNA) sequencing data generated in a previous study based on the HELIUS cohort [3]. In short, the composition of fecal microbiota was profiled by sequencing the V4 region of the 16S rRNA gene on a MiSeq system. The 16S rRNA gene reads were processed on a mothur pipeline (version 1.39.5) [12]. The OTU clustering was done by using the vsearch (version 2.6) [13] and a phylogenetic tree was constructed by running FastTree 2.1 [14]. The details of the sequencing and bioinformatic pipelines were described in [3].

### Statistical analyses

Our analysis is based on 1090 subjects who had both fecal microbiome and FFQ data. Following [1], here we removed OTUs with fewer than 10 reads in total, as well as OTUs which were present in fewer than 1% of samples. The final OTU table contains 1090 samples and 2073 OTUs. We used four widely used methods for sequencing data analysis to quantify the strength of the associations between dietary variables (*x*) and OTU counts (*y*). Because the large number of associations (67 × 2073), we used multidplyr R package (https://github.com/hadley/multidplyr) for parallel computation. The selected methods were as follows:

ShotgunFunctionalizeR is a popular R package used in microbiome research community, and based on the Poisson generalized linear model (implemented in glm function in R) [15]. We used the glm function with log(total counts) as offset to quantify associations between dietary variables and OTU counts.

Negative binomial model, also called gamma-Poisson model, is popular for statistical modeling of OTU count data [16, 17]. Phyloseq is a popular R package used by the microbiome research community [18]. The core of Phyloseq is based on another popular R package DESeq2, which is based on negative binomial model [19]. However, when the sample size is big (above 100), the computation becomes slow in DESeq2. Therefore, in this work we used another negative binomial based R package, edgeR [20]. The observed OTU count was modeled by a negative binomial distribution with two parameters, the mean and the dispersion. OTU specific dispersion was estimated by running estimateDisp function implemented in the edgeR package [20, 21]. The associations between dietary variables and OTU counts were quantified by running glmFit function of the edgeR package [20]. The log(total counts) was used as offset.

In contrast to above methods modeling the counts with exact probabilistic distributions, others have advocated weighted linear regression analysis with precision weights derived from the mean variance relationship [22]. This approach has been implemented in the voom function of the popular R package limma [23]. The weighted linear regression was done to estimate the linear association between dietary variables and OTU counts with precision weights estimated by the voom and lmFit functions in the limma package [22, 23].

The last method, metagenomeSeq is also a popular R package used by microbiome research community [24]. It is based on the zero-inflated Gaussian model. This approach has been implemented in the fitZig function of the popular R package metagenomeSeq [24]. The cumulative sum scaling method was used to take care library size difference.

In a typical association study, the primary goal is to identify some candidate associations for future research. Therefore, regarding multiple testing we calculated false discovery rate (FDR). If an association had FDR value below 0.05, we considered it as a significant association. Since the research question is focused only on robustness of the statistical results and not on biological or epidemiological associations, we did not adjust for possible confounding or selection factors.

### Simulation framework

We use *y* to represent the simulated microbiome data with *n* rows and *J* columns. Every column of *y* represents a microbe and every row of *y* represents a subject. Here, we simulated associations of a dietary variable, denoted as *x*, with gut microbiota. *x* is a vector of length *n*, and was randomly sampled from real FFQ data with replacement. The FFQ data was published in [2] and contained 214 dietary variables that were scaled to having mean 0 and standard deviation 1. For each simulated microbiome data set, we used one dietary variable and in total generated 214 simulated data sets. Our simulation framework included the steps below:

1 $$ \eta \left[j\right]\sim Bernoulli(0.5) $$

2 $$ \gamma \left[j\right]\sim {T}_7\left(0,2.5\right) $$

3 $$ \beta \left[j\right]=\left(1-\eta \left[j\right]\right)\times 0+\eta \left[j\right]\times \gamma \left[j\right] $$

4 $$ \theta \left[i,1:J\right]\sim Dirichlet\left(\pi \left[1:J\right]\right) $$

5 $$ \alpha \left[i,1:J\right]= logit\left(\theta \left[i,1:J\right]\right) $$

6 $$ logit\left(\mu \left[i,j\right]\right)=\alpha \left[i,j\right]+\beta \left[j\right]\times x\left[i\right] $$

7 $$ N\left[i\right]\sim Lognormal\left({\mu}_L,{\sigma}_L\right) $$

8 $$ y\left[i,1:J\right]\sim Multinomial\left(N\left[i\right],\mu \left[i,1:J\right]\right) $$

Our HELIUS microbiome data set had 1090 subjects and the median sequencing depth was about 50,000. To mimic HELIUS data, we simulated the 16S microbiome data sets, with each data set having 1000 subjects and mean of sequencing depth 50,000.

### Spiked-in association

To introduce the spiked-in association between a dietary variable and microbe *j*, we need two variables *η*[*j*] and *γ*[*j*]. The indicator variable, *η*[*j*], indicates if a dietary variable influences the abundance of the microbe *j*. For microbe *j*, we randomly drew *η*[*j*] from a Bernoulli distribution with parameter 0.5 (Eq. 1). *γ*[*j*] represents the effect of the dietary variable on the abundance for OTU *j*, and was sampled from a t distribution with 7 degrees of freedom, location 0 and scale 2.5 [25] (Eq. 2). Then the true association between the diet and microbe *j* was captured by *β*[*j*] defined in Eq. 3.

### Dirichlet multinomial model

In the current study, we developed a Dirichlet multinomial model to generate 16S rRNA microbiome data.

In Eq. 4, the matrix *θ* has *n* rows and *J* columns. *θ*[*i*, *j*] corresponds to the baseline abundance level for the microbe *j* in subject *i*. For subject *i*, we randomly drew a vector of length *J* from a Dirichlet distribution. In Eq. 5, the parameter of the Dirichlet distribution *π* is a vector of length *J*. We used R package DirichletMultinomial [26] and the Human Microbiome Project 16S rRNA stool data [27] to estimate the *π*. In Eq. 6, the true microbe *j* proportion in subject *i*, *μ*[*i*, *j*] was modeled as a logistic regression of *x*[*i*]. Similar to [24], library size of subject *i*, *N*[*i*], was randomly drawn from a lognormal distribution with mean *μ*_*L*_ and standard deviation *σ*_*L*_ (Eq. 7). *μ*_*L*_ is the logarithm of target sequencing depth (50000). We estimated *σ*_*L*_ = 0.77 based on the HMP stool 16S rRNA data set by using the fitdistr function implemented in the MASS package. Finally, for subject *i*, the observed microbe counts were randomly generated from a multinomial distribution (Eq. 8).

### Performance evaluation

We evaluated the model performances based on metrics including true positive rate, false positive rate and error probability for identifying a significant association between microbe and dietary variable. They are calculated per simulation and defined as below:

9 $$ \mathrm{True}\ \mathrm{positive}\ \mathrm{rate}=\frac{\mathrm{TP}}{\mathrm{TP}+\mathrm{FN}} $$

10 $$ \mathrm{False}\ \mathrm{positive}\ \mathrm{rate}=\frac{\mathrm{FP}}{\mathrm{TN}+\mathrm{FP}} $$

11 $$ \mathrm{Error}\ \mathrm{probability}=\frac{FP}{\mathrm{TP}+\mathrm{FP}} $$

TP, FP, TN and FN refer to true positive, false positive, true negative and false negative, respectively. The indicator variable, *η*[*j*], is in the definition of our spike-in associations. When *η*[*j*] = 1, the dietary variable *x* influences the abundance of the microbe *j*, otherwise *η*[*j*] = 0. Therefore, a true positive finding is defined as having a significant association between the dietary variable *x* and microbe *j* with FDR < 0.05 in case the true *η*[*j*] = 1. A false positive finding is defined as having a significant association between the dietary variable *x* and microbe *j* with FDR < 0.05 in case the true *η*[*j*] = 0. A true negative finding is defined as having a association between the dietary variable *x* and microbe *j* with FDR > 0.05 in case the true *η*[*j*] = 0. A false negative finding is defined as having a association between the dietary variable *x* and microbe *j* with FDR > 0.05 in case the true *η*[*j*] = 1. The error probability quantified the probability that a significant association is false. Here we did not use “false discovery rate” but used the term “error probability” in order to avoid confusion, because we also calculated the false discovery rate during analyses of associations between OTUs and dietary variables.

## Abbreviations

FDR: False discovery rate
FFQs: Food frequency questionnaires
FN: False negative
FP: False positive
HELIUS: HEalthy Life in an Urban Setting
OTU: Operational taxonomic unit
TN: True negative
TP: True positive
